# Mapping the structure of depression biomarker research: A bibliometric analysis

**DOI:** 10.3389/fpsyt.2022.943996

**Published:** 2022-09-16

**Authors:** Xiang-jie Guo, Peng Wu, Xiao Jia, Yi-ming Dong, Chun-mei Zhao, Nian-nian Chen, Zhi-yong Zhang, Yu-ting Miao, Ke-ming Yun, Cai-rong Gao, Yan Ren

**Affiliations:** ^1^Department of Forensic Medicine, Shanxi Medical University, Taiyuan, China; ^2^College of Pharmacy, Nankai University, Tianjin, China; ^3^Department of Health Statistics, School of Public Health, Shanxi Medical University, Taiyuan, China; ^4^Department of Psychology, School of Humanities and Social Sciences, Shanxi Medical University, Taiyuan, China; ^5^Department of Psychiatry, Shanxi Bethune Hospital, Shanxi Academy of Medical Sciences, Tongji Shanxi Hospital, Third Hospital of Shanxi Medical University, Taiyuan, China; ^6^Tongji Hospital, Tongji Medical College, Huazhong University of Science and Technology, Wuhan, China

**Keywords:** depression, biomarker, bibliometric analysis, co-word analysis, co-citation analysis

## Abstract

**Background:**

Depression is a common mental disorder and the diagnosis is still based on the descriptions of symptoms. Biomarkers can reveal disease characteristics for diagnosis, prognosis, and treatment. In recent years, many biomarkers relevant to the mechanisms of depression have been identified. This study uses bibliometric methods and visualization tools to analyse the literature on depression biomarkers and its hot topics, and research frontiers to provide references for future research.

**Methods:**

Scientific publications related to depression biomarkers published between 2009 and 2022 were obtained from the Web of Science database. The BICOMB software was used to extract high-frequency keywords and to construct binary word-document and co-word matrices. gCLUTO was used for bicluster and visual analyses of high-frequency keywords. Further graphical visualizations were generated using R, CiteSpace and VOSviewer software.

**Results:**

A total of 14,403 articles related to depression biomarkers were identified. The United States (34.81%) and China (15.68%), which together account for more than half of all publications, can be considered the research base for the field. Among institutions, the University of California, University of London, and Harvard University are among the top in terms of publication number. Three authors (Maes M, Penninx B.W.J.H., and Berk M) emerged as eminent researchers in the field. Finally, eight research hotspots for depression biomarkers were identified using reference co-citation analysis.

**Conclusion:**

This study used bibliometric methods to characterize the body of literature and subject knowledge in the field of depression biomarker research. Among the core biomarkers of depression, functional magnetic resonance imaging (fMRI), cytokines, and oxidative stress are relatively well established; however, research on machine learning, metabolomics, and microRNAs holds potential for future development. We found “microRNAs” and “gut microbiota” to be the most recent burst terms in the study of depression biomarkers and the likely frontiers of future research.

## Introduction

Depression is a common and serious mental disorder characterized by the presence of sadness and loss of interest, possibly leading to suicide. The World Health Organization reports that by 2030, depression will be the leading cause of disease burden, with a global prevalence ranging from 3 to 17% (1, 2). The treatment options are currently diverse and include medication, psychotherapy and physiotherapy, but the most common clinical treatment for depression continues to be antidepressant (AD) medication (3). However, approximately one-third of patients with depression do not respond to existing antidepressants (4). Given the clinical heterogeneity of depression, the identification of biomarkers that can improve the diagnosis and classification of depression or predict the effectiveness of medications has always been a goal of clinicians (5, 6).

Biomarkers are measurable characteristics of an individual that may represent risk factors for a disease or outcome or may be indicators of disease progression or treatment-associated changes (7). They are commonly classified as diagnostic, predictive, or moderators. Research over the past two decades has shown that neuroimaging, neurophysiology, genomics, proteomics, and metabolomics can provide candidate biomarkers and pointed to their importance for discriminating depression subtypes and working toward precision medicine (8).

Bibliometrics is a statistical analysis method that identifies particularly impactful papers, research hotspots, and future emerging trends through co-word and co-citation analyses of publications within a specific research field (9, 10). Some studies have been conducted using bibliometric methods to analyze characteristics of literature related to the field of depression. Xu et al. analyzed the depression-related literature in Web of Science database from 1998 to 2018 to provide a systematic summary of the etiology, mechanisms, and treatment of depression (11). Zou et al. complemented the association between depression and insulin using a bibliometric approach (12). Zhu et al. performed a bibliometric analysis of depression and gut microbes (13). Xiang et al. used a bibliometric approach to gain insight into the potential of acupuncture for the treatment of depression (14). You et al. used a bibliometric approach to characterize the literature on exercise interventions for depression among adolescents over the last two decades (15). However, bibliometric analyses on biomarkers of depression have rarely been reported. In this study, we collected scientific publications related to biomarkers of depression from the Web of Science (WoS) database from 1 January 2009 to 10 March 2022. R, CiteSpace, VOSviewer, BICOMB, and BibExcel software were used to summarize existing studies and explore the knowledge structure of the field. Our goal was to provide a systematic overview of current scientific achievements and future trends in depression biomarker research.

## Methods

### Search terms and retrieval strategies

We conducted a literature search in the Web of Science Core Collection (WoSCC) (Clarivate Analytics, Philadelphia, Pennsylvania, USA), which covers a large number of records and documents. The final retrieval strategies were as follows: subject words = (depression ^*^) or (depressive disorder ^*^) or (dysthymia ^*^) or (major depressive disorder ^*^) and (biomarker ^*^) or (biomarkers ^*^) or (marker ^*^) or (markers ^*^); literature type = article; language type = English. Records and references of 14,403 studies were downloaded in TXT format. To avoid deviations due to database updates, the process of literature search and data extraction was conducted once on 10 March 2022 and the results were subsequently imported into bibliometric analysis tools for analysis. In addition, we used the search strategy of “(psychosis^*^) and ”(biomarker^*^) or (biomarker^*^) or (marker^*^) or (marker^*^)” and performed a bibliographic analysis using the same methodology to compare the similarities between psychosis and depression in terms of biomarkers.

### Co-citation analysis

CiteSpace is a freely available Java-based application developed by Dr. Chaomei Chen for visualizing and analyzing trends and patterns in scientific literature. Citation analysis is an important feature of CiteSpace that predicts the impact of publications in a specific field of research. The following parameters were used for analysis: from 2009 to 2022, slice = 1, and the top 50 most cited papers per year per individual network. Popular citations within the field of research are represented by nodes in the network, with larger nodes indicating more frequent citations (16, 17). We derived the literature co-citation network and performed keyword-burst detection. The dual-map was also executed by citespace. Additionally, VOSviewer was used to visualize collaborative networks and keyword co-occurrence between countries/regions and institutions, as well as co-citations of authors (18).

### Co-word analysis

BICOMB was used as a text mining tool to extract high-frequency keywords from the TXT files and to generate the binary matrix (19). We then performed a bicluster analysis on this matrix using gCLUTO to identify hotspots of research on biomarkers of depression (20, 21). The results were visualized in mountain and heat map representations. In addition, the Bibliometrix package, a bibliometric analysis tool based on the R language, was used to depict the source dynamics of journals and core author and journal association.

## Results

### Annual analysis of publications

**Figure 1:**
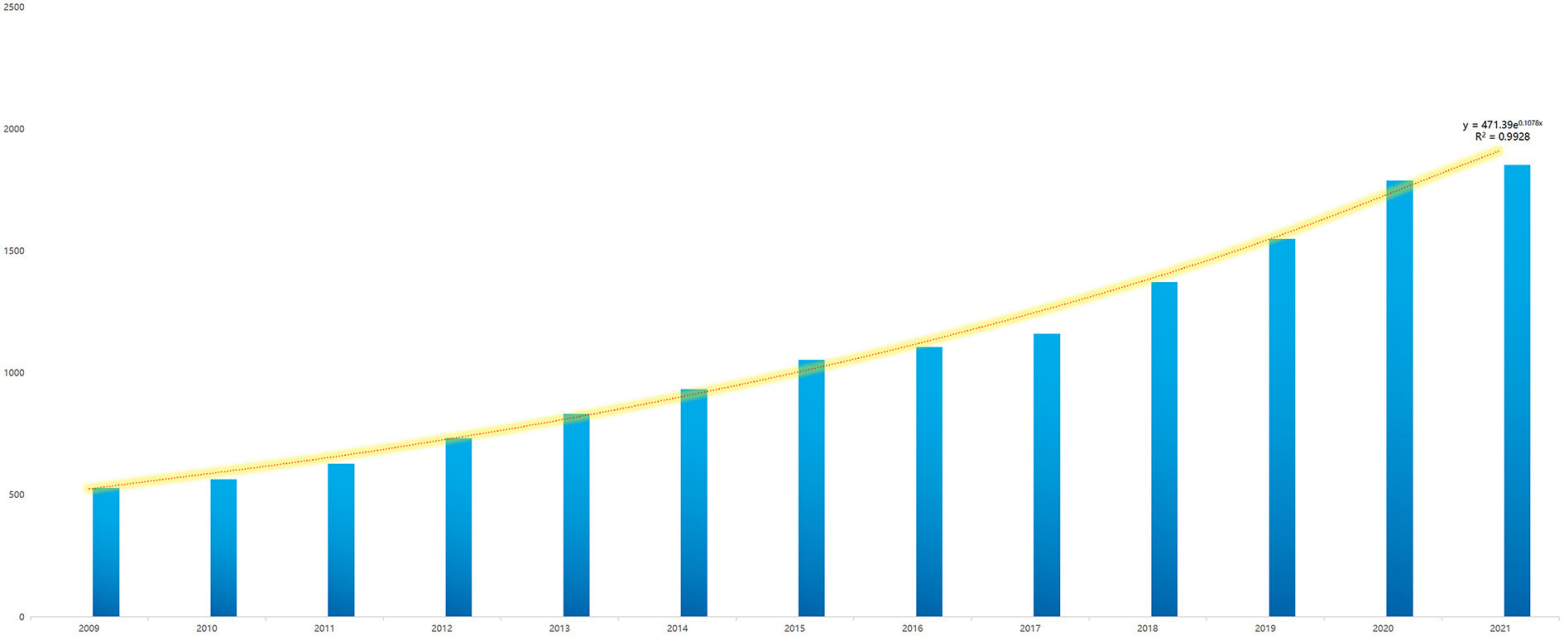
Annual number of publications in depression biomarker research from 2009 to 2022.

A total of 14,403 depression biomarker articles were published in WoS between 2009 and 10 March 2022 (Figure 1). The cumulative number of articles related to biomarkers of depression has been increasing rapidly since 2009. The respective numbers of publications per year are: 530 publications in 2009, 564 in 2010, 629 in 2011, 733 in 2012, 834 in 2013, 934 in 2014, 1,055 in 2015, 1,108 in 2016, 1,161 in 2017, 1,373 in 2018, 1,550 in 2019, 1,790 in 2020 and 1,854 in 2021. Notably, documents of 2022 were included only from 1 January to 10 March, a total of 288 papers, which is only 2% of the overall. The model fitted to the annual growth data, y = 471.39e^0.1078X^ (*R*^2^ = 0.9928), predicts that the number of papers published in this field in 2022 will be 2,132. This suggests that the known depression biomarkers are increasing in number and have clinical significance and developmental potential.

### Distribution characteristics of countries/regions and institutions

**Figure 2:**
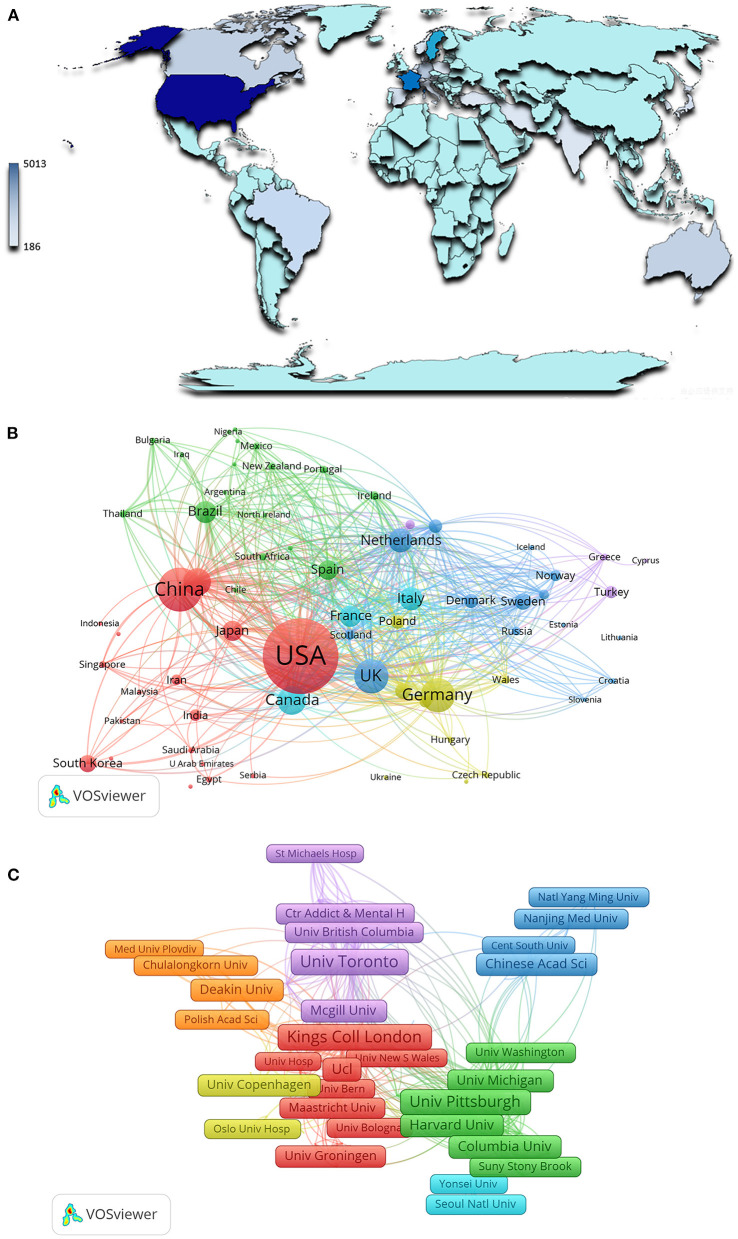
Main countries/regions and institutions of depression biomarker research and their interrelationships. **(A)** Countries/Regions distribution of depression biomarker research results; **(B)** A visualization network of collaboration between countries/regions in depression biomarker research; **(C)** A visualization network of collaboration among institutions in depression biomarker research. The nodes in the map denote elements such as a country or institute, and link lines between nodes denote collaborative relationships. The larger the circle/frame, the more articles are published. The wider the line, the stronger the relationship.

Between 2009 and 2022, 137 countries and regions published studies related to biomarkers of depression. The contributing countries or regions are displayed on a graph generated using CiteSpace and Google Earth (Figure 2). Among the top 10 contributing countries, The United States had the highest number of publications (*n* = 5,002), followed by China (*n* = 2,025), Germany (*n* = 1,333), and the United Kingdom (*n* = 1,321) (Table 1). As can be seen from the cooperation network of countries and regions, the network nodes of the USA, China, UK, and Germany are not only located at the center of the cooperation relationship map but also have the strongest links with the other major issuing countries (Figure 2B).

**Table 1:** The main countries, regions, and institutions contributing to publications in depression biomarker research.

**Rank**	**Country/region**	**Article counts**	**Proportion**	**Institutions**	**Article counts**	**Proportion**	**H index**	**Total number of citations**	**Average number of citations**
1	The United States	5,002	34.73%	University of California System	702	4.87%	76	22,355	31.84
2	China	2,025	14.06%	University of London	626	4.35%	79	23,866	38.12
3	Germany	1,333	9.26%	Harvard University	579	4.02%	71	22,281	38.48
4	United Kingdom	1,321	9.17%	The Pennsylvania State System of Higher Education	409	2.84%	61	13,367	32.68
5	Canada	996	6.92%	University of Toronto	372	2.58%	48	10,678	28.7
6	Australia	928	6.44%	King's College London	370	2.57%	65	15,382	41.57
7	Netherlands	740	5.14%	US Department of Veterans Affairs	369	2.56%	59	13,104	35.51
8	Italy	702	4.87%	Veterans Health Administration	359	2.49%	58	12,788	35.62
9	Brazil	633	4.39%	National Institutes of Health	336	2.33%	63	15,965	47.51
10	France	608	4.22%	University of Texas System	324	2.25%	47	8,303	25.63

In the last decade, 11,954 institutions have published research related to depression biomarkers. The top 10 contributing institutions published 4,446 articles, accounting for 37.2% of the total (Table 1). Among them, the University of California System contributed the most to the body of knowledge (*n* = 702), followed by the University of London (*n* = 626), Harvard University (*n* = 579), and the Pennsylvania Commonwealth System of Higher Education (*n* = 409). We analyzed the collaboration network between institutions using VOSviewer (Figure 2C).

### Journal analysis

**Figure 3:**
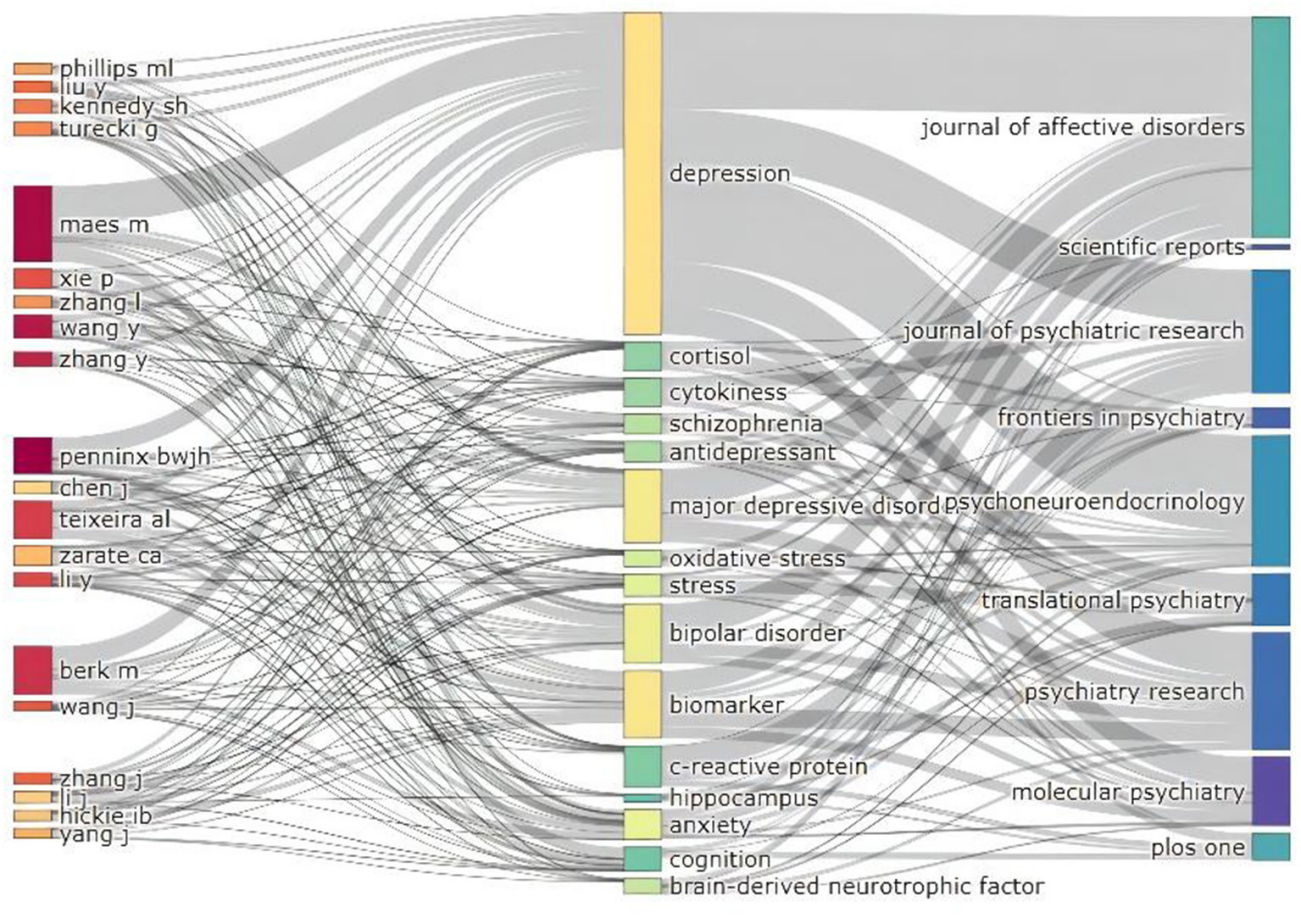
Three-field plot of active institutions and authors publishing articles related to depression biomarkers between 2009 and 2022.

A total of 2,362 journals have published articles on the biomarkers of depression since 2009. We identified the top 10 contributing journals (Table 2), which together published 2,803 articles, representing 20.31% of all publications on depression biomarkers. Therefore, by considering articles published in these journals, we can obtain an overview of the research frontiers in the field. *Brain Behavior and Immunity* and *Psychiatry Research* are not only journals with a high number of published papers, but also those with a high impact factor in this field. They appear to be an important source of knowledge on biomarkers of depression. The Sankey diagram links authors, keywords and sources, and our analysis reveals a number of authors and journals that have made significant contributions to the field of biomarkers of depression (Figure 3).

**Table 2:** The top 10 highly-productive journals in depression biomarker research.

**Rank**	**Journal**	**Number of publications**	**Proportion**	**IF (2022)**	**Quartile in category (2022)**
1	Journal of Affective Disorders	609	4.23%	6.533	Q1
2	Plos One	376	2.61%	3.752	Q2
3	Brain Behavior and Immunity	272	1.89%	19.227	Q1
4	Psychoneuroendocrinology	268	1.86%	4.693	Q2
5	Journal of Psychiatric Research	265	1.84%	5.250	Q2
6	Translational Psychiatry	245	1.70%	7.989	Q1
7	Psychiatry Research	215	1.49%	11.225	Q1
8	Frontiers in Psychiatry	210	1.46%	5.435	Q2
9	Scientific Reports	204	1.42%	4.996	Q2
10	Molecular Psychiatry	139	0.97%	13.437	Q1

**Figure 4:**
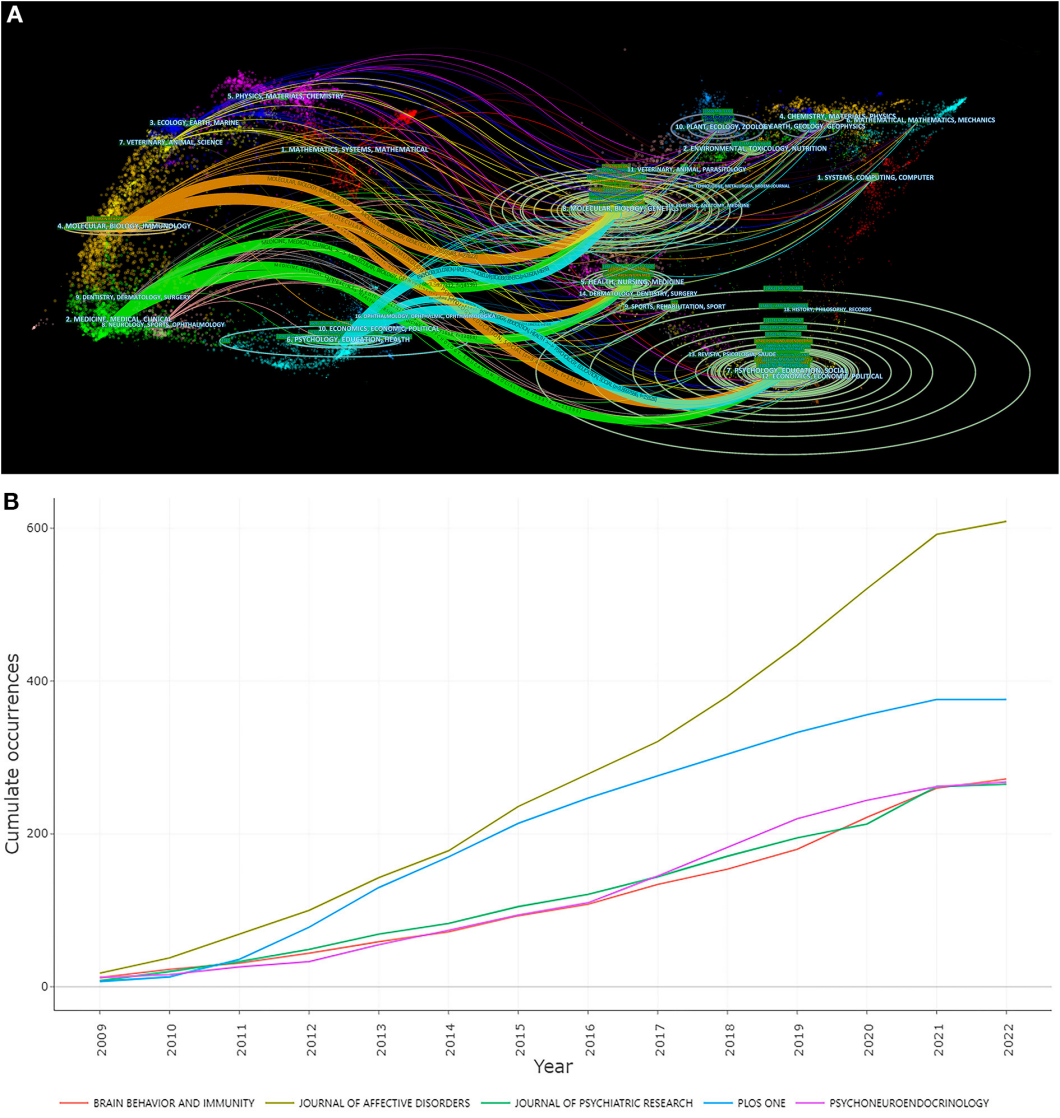
The visualization of Core Journals. **(A)** Biplot overlay of article citations for depression biomarker research. **(B)** Source dynamics of top 5 Core Journals.

The “journal biplot” overlay was used to reveal the trends in the scientific literature at a global level. A citation overlay was created using CiteSpace's bipartite overlay feature to visualize citations of studies on depression biomarkers. In the resulting figure, the citing graph is on the left, the cited graph is on the right, and the curves are citation pathways connecting the lines from left to right. We identified five major citation paths in the biplot overlay of the journals. The results suggest that studies published in MEDICINE|MEDICAL|CLINICAL, MOLECULAR|BIOLOGY|IMMUNOLOGY and PHYSICS|EDUCATION|HEALTH are frequently cited (Figure 4A). Between 2009 and 2022, the number of articles published in these journals showed a year-on-year increase, with *The Journal of Affective Disorders* showing the most significant growth rate (Figure 4B). The analysis of the core author and intellectual basis of the research field is shown in Supplementary material (Supplementary Figure 1; Supplementary Table 1).

### Research hotspots: Clustering analysis of keywords and co-word analysis

According to the keyword clustering map and hotspot density map, the research theme of depression biomarkers was divided into five clusters (Supplementary Figure 2). In the first cluster (red), “depression” appears most frequently as the main keyword, followed by “inflammation”, “stress”, “anxiety”, “interleukine-6”, “C-reactive protein”, and “cortisol”. In the second cluster (green), the most frequently used keyword for this cluster is “biomarkers”, with other high-frequency keywords including “major depressive disorder”, “fMRI”, “functional connectivity”, and “genome-wide association”. In the third cluster (blue), the keywords for the “brain” appear most frequently, followed by “neuroinflammation”, “expression”, “brain-derived neurotrophic factor (BDNF)”, “hippocampus”, and “HPA axis”. The fourth cluster (yellow) includes “dementia”, “diagnosis”, “dysfunction”, and “mild cognitive impairment”. The fifth cluster (purple) contains only three keywords, including “inbreeding”, “depressions”, and “microsatellites”.

**Figure 5:**
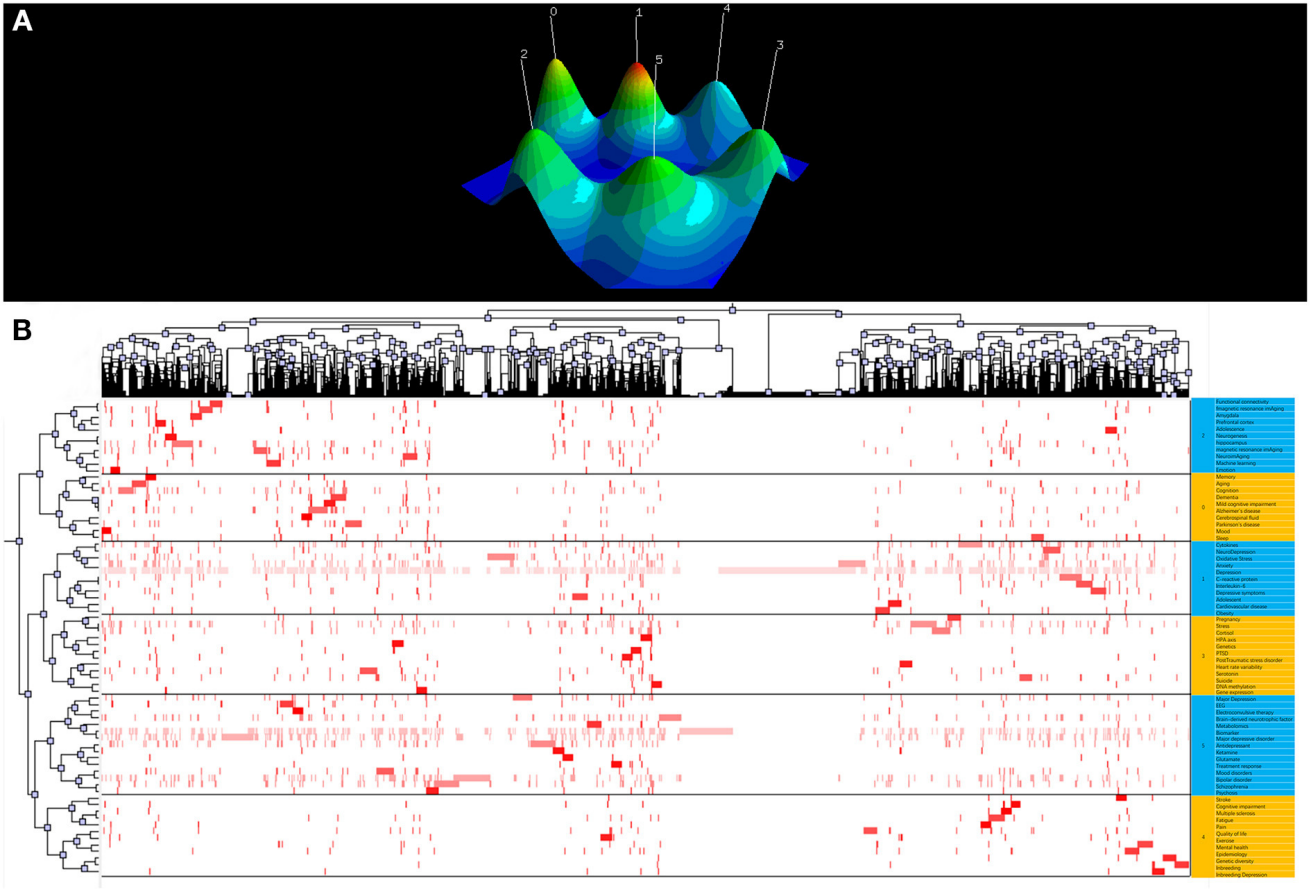
The visualization of keywords biclustering analysis of depression biomarker research. **(A)** Visualized Mountain Map based on the Biclustering analysis of depression biomarker Binary Matrix of Word-paper. **(B)** Visualized matrix based on the biclustering analysis of depression biomarker binary matrix of Word-paper.

In this study, 25,354 keywords were extracted by BICOMB. According to the H-index, a keyword can be defined as high frequency when its frequency is equal to or greater than its ordinal number (Table 3). A binary matrix (Table 4) was constructed for high-frequency keywords, and we performed a biclustering analysis using gCLUTO to create a hill and heat map (Figure 5).

**Table 3:** Top 70 high-frequency keywords in depression biomarker research.

**Rank**	**Keywords**	**Frequency, *n***	**Percentage, %**	**Cumulative percentage, %**
1	Depression	3,993	6.0695	6.0695
2	Biomarker	1,207	1.8347	7.9042
3	Major depressive disorder	760	1.1552	9.0594
4	Bipolar disorder	584	0.8877	9.9471
5	Anxiety	558	0.8482	10.7953
6	Stress	383	0.5822	11.3775
7	Oxidative Stress	347	0.5275	11.9049
8	Brain-derived neurotrophic factor	339	0.5153	12.4202
9	Schizophrenia	330	0.5016	12.9218
10	Antidepressant	329	0.5001	13.4219
11	Cytokines	322	0.4895	13.9114
12	Cortisol	272	0.4134	14.3248
13	Cognition	255	0.3876	14.7124
14	C-reactive protein	255	0.3876	15.1000
15	Hippocampus	250	0.3800	15.4800
16	Major depression	238	0.3618	15.8418
17	Alzheimer's disease	215	0.3268	16.1686
18	Mood disorders	176	0.2675	16.4361
19	Parkinson's disease	174	0.2645	16.7006
20	Suicide	170	0.2584	16.9590
21	Neuro depression	168	0.2554	17.2144
22	Serotonin	163	0.2478	17.4622
23	Interleukin-6	161	0.2447	17.7069
24	Quality of life	150	0.2280	17.9349
25	fMRI	147	0.2234	18.1583
26	Aging	146	0.2219	18.3803
27	Sleep	145	0.2204	18.6007
28	Depressive symptoms	141	0.2143	18.8150
29	Adolescent	136	0.2067	19.0217
30	Neuroimaging	136	0.2067	19.2284
31	Mental health	135	0.2052	19.4336
32	Fatigue	134	0.2037	19.6373
33	Dementia	132	0.2006	19.8380
34	EEG	123	0.1870	20.0249
35	Functional connectivity	123	0.1870	20.2119
36	Pregnancy	122	0.1854	20.3973
37	Obesity	122	0.1854	20.5828
38	Epidemiology	121	0.1839	20.7667
39	Inbreeding	118	0.1794	20.9461
40	Machine learning	117	0.1778	21.1239
41	Metabolomics	111	0.1687	21.2926
42	Amygdala	110	0.1672	21.4598
43	Genetic diversity	100	0.1520	21.6118
44	Adolescence	98	0.1490	21.7608
45	Magnetic resonance imaging	97	0.1474	21.9083
46	Cardiovascular disease	94	0.1429	22.0511
47	Heart rate variability	93	0.1414	22.1925
48	Exercise	91	0.1383	22.3308
49	Psychosis	90	0.1368	22.4676
50	Prefrontal cortex	90	0.1368	22.6044
51	Mild cognitive impairment	89	0.1353	22.9936
52	Post-traumatic stress disorder	89	0.1353	23.1288
53	Inbreeding depression	89	0.1353	23.2641
54	PTSD	87	0.1322	23.3964
55	DNA methylation	85	0.1292	23.5256
56	Gene expression	84	0.1277	23.6532
57	Glutamate	82	0.1246	23.7779
58	Genetics	82	0.1246	23.9025
59	Ketamine	82	0.1246	24.0272
60	Neurogenesis	82	0.1246	24.1518
61	HPA axis	80	0.1216	24.2734
62	Treatment response	80	0.1216	24.3950
63	Stroke	78	0.1186	24.5136
64	Cognitive impairment	77	0.1170	24.6306
65	Pain	75	0.1140	24.7446
66	Multiple sclerosis	74	0.1125	24.8571
67	Electroconvulsive therapy	73	0.1110	24.9681
68	Memory	73	0.1110	25.0790
69	Cerebrospinal fluid	71	0.1079	25.1870
70	Mood	71	0.1079	25.2949
71	Emotion	70	0.1064	25.4013

**Table 4:** Binary matrix table of depression biomarker high-frequency keywords and articles.

**NO**.		**Paper ID**					
**Keyword**		**001**	**002**	**003**	**…**	**14402**	**14403**
1	Depression	1	0	1	…	0	0
2	Biomarker	0	1	0	…	0	0
3	Major depressive disorder	1	1	0	…	0	0
4	Bipolar disorder	0	1	0	…	0	0
5	Anxiety	0	0	0	…	0	0
…	…	…	…	…	…	…	…
70	Cerebrospinal fluid	0	0	0	…-	0	0
71	Emotion	0	0	0	…	0	0

**Figure 6:**
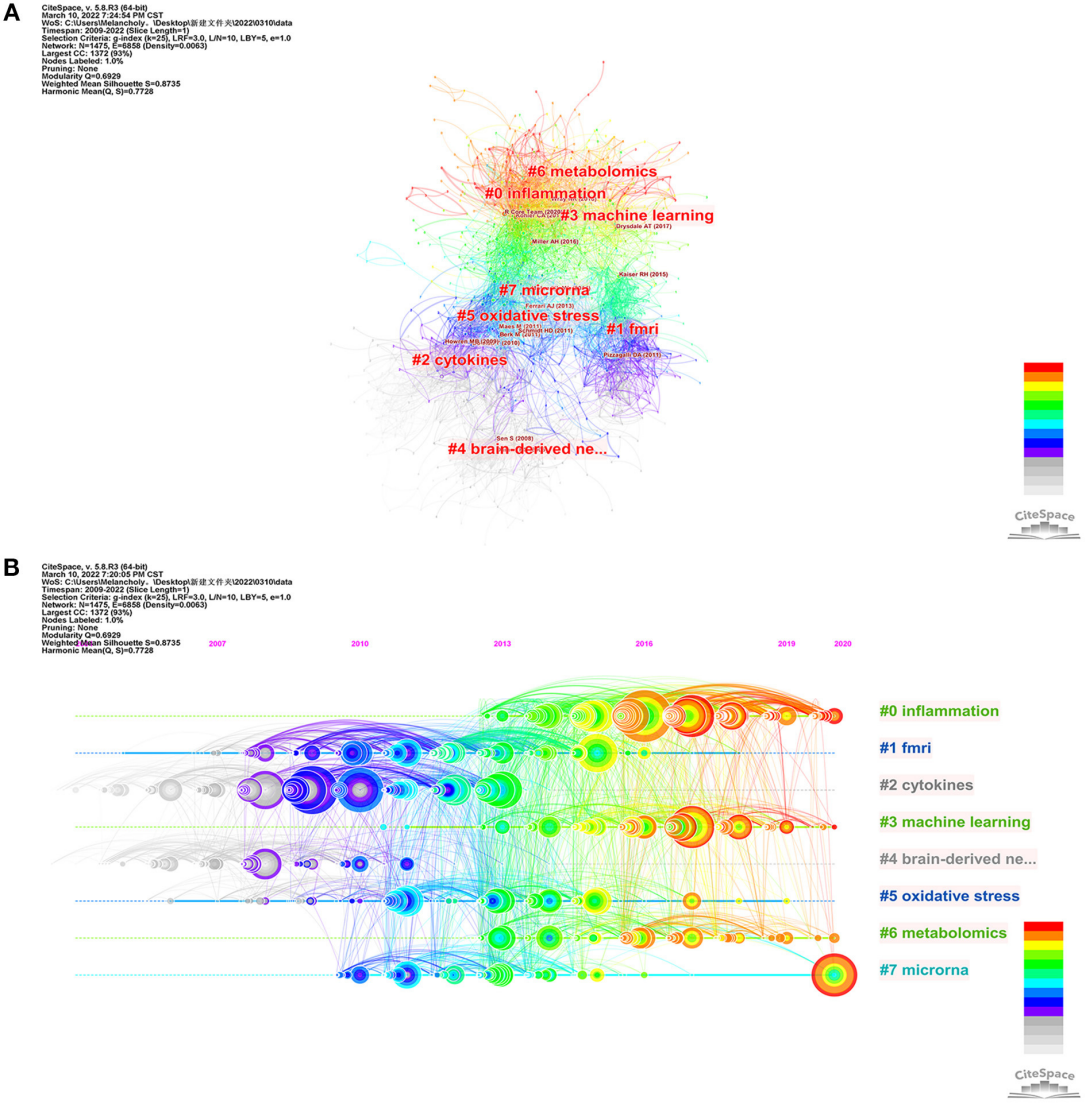
The visualization of reference co-citation analysis of depression biomarker research. **(A)** Co-citation network mapping of literature related to depression biomarker research. **(B)** Timeline related to depression biomarker studies. The nodes in the map denote co-cited references, and links between nodes denote co-citation relationships. The citation rings denote the citation history of a reference. Large nodes or nodes with red tree-rings are either highly cited or have citation bursts in a given time slice.

Reference co-citation analysis (RCA) is used to explore research hotspots in a given field. Based on specific metrics, term frequency–inverse document frequency, log-likelihood tests (LLR), and mutual information tests, CiteSpace can extract noun phrases from the titles of articles to characterize clusters. Previous studies have shown that LLR labels delivered the best results for covering topics (Table 5). Silhouette values > 0.7 indicate high confidence in the clusters. Articles published between 2009 and 2022 related to biomarkers of depression were grouped into eight main clusters including “inflammation”, “fMRI”, “cytokines”, “machine learning”, “brain-derived neurotrophic factor”, “oxidative stress”, “metabolomics”, and “microRNAs”. A timeline view was drawn using CiteSpace for all clusters (Figure 6). The publication date is at the top, with more recent publications located further to the right. The results show that “cytokines” appeared first and “metabolomics” last, with current research focusing more on “inflammation” and “microRNAs”, which may be the focus of future research. In addition, to explore the links between depression and other brain disorders, we adopted the same strategy to analyse psychosis and biomarker bibliographic data and found some similarities between the two in terms of inflammation and imaging (Supplementary Figures 3, 4).

**Table 5:** The largest 8 clusters of depression biomarkers references co-citation, identified by subject headings.

**Cluster ID**	**Size**	**Silhouette**	**Mean (cite Year)**	**Label (LSI)**	**Label (LLR)**	**Label (MI)**
0	245	0.787	2017	Major depressive disorder	Inflammation	Adjunctive therapy
1	191	0.862	2011	Bipolar disorder	fMRI	Emotional processing bias
2	166	0.901	2008	c-reactive protein	Cytokines	Depression symptom dimensions
3	143	0.876	2016	Aging effect	Machine learning	Default mode network
4	136	0.889	2007	Brain-derived neurotrophic factor	Brain-derived neurotrophic factor	Nestin
5	109	0.874	2012	Gene expression	oxidative stress	Interleukin-1 receptor antagonist
6	105	0.869	2016	Risk prediction	Metabolomics	Cfs
7	98	0.862	2012	Cost effectiveness analysis	Microrna	Early nutrition

LSI, Latent semantic indexing; LLR, log-likelihood ratio; MI, mutual information.

### Research frontiers: Citations and keywords burst analysis

**Figure 7:**
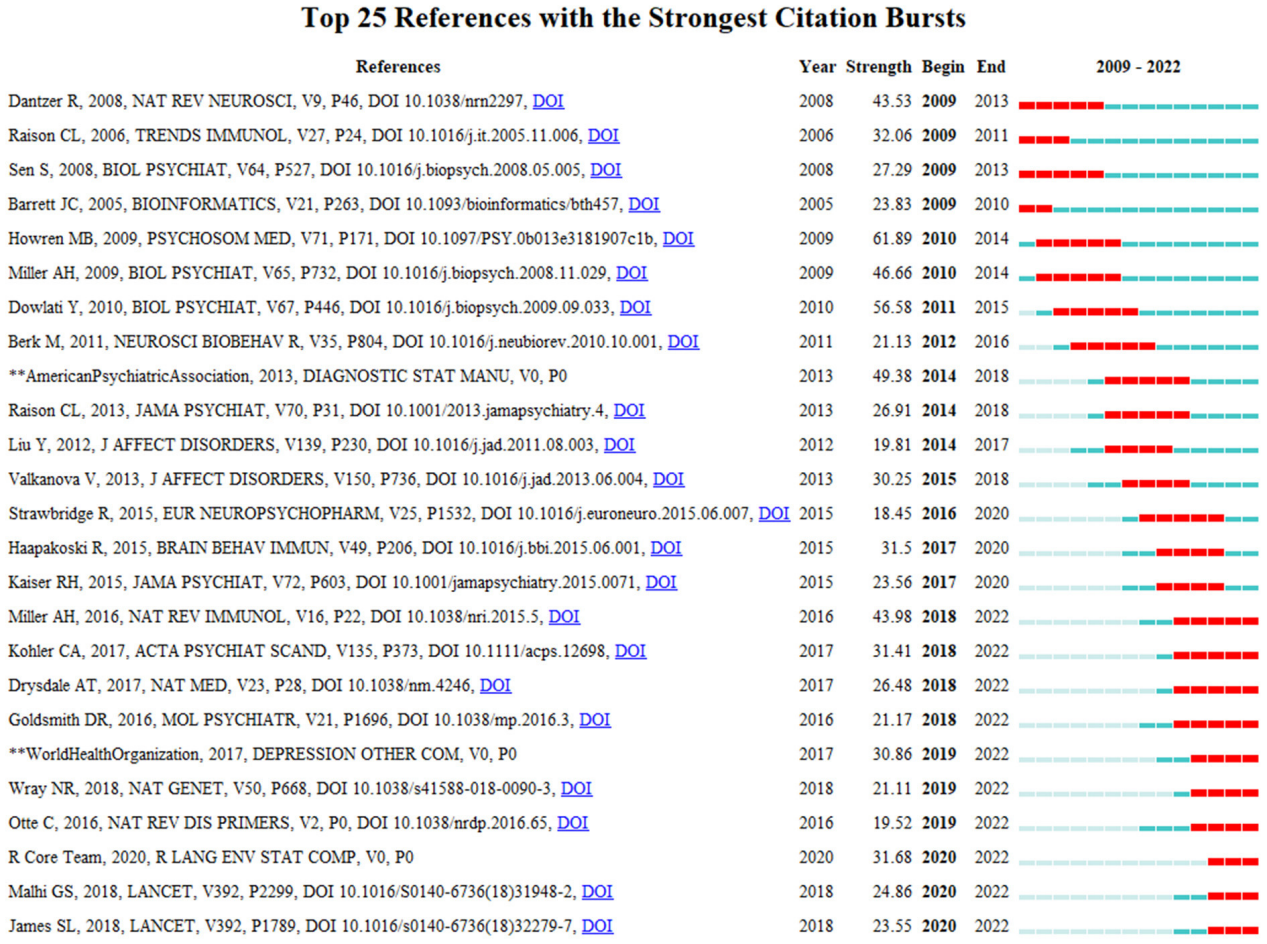
Top 25 citings with the strongest citation bursts, 2009–2022.

**Figure 8:**
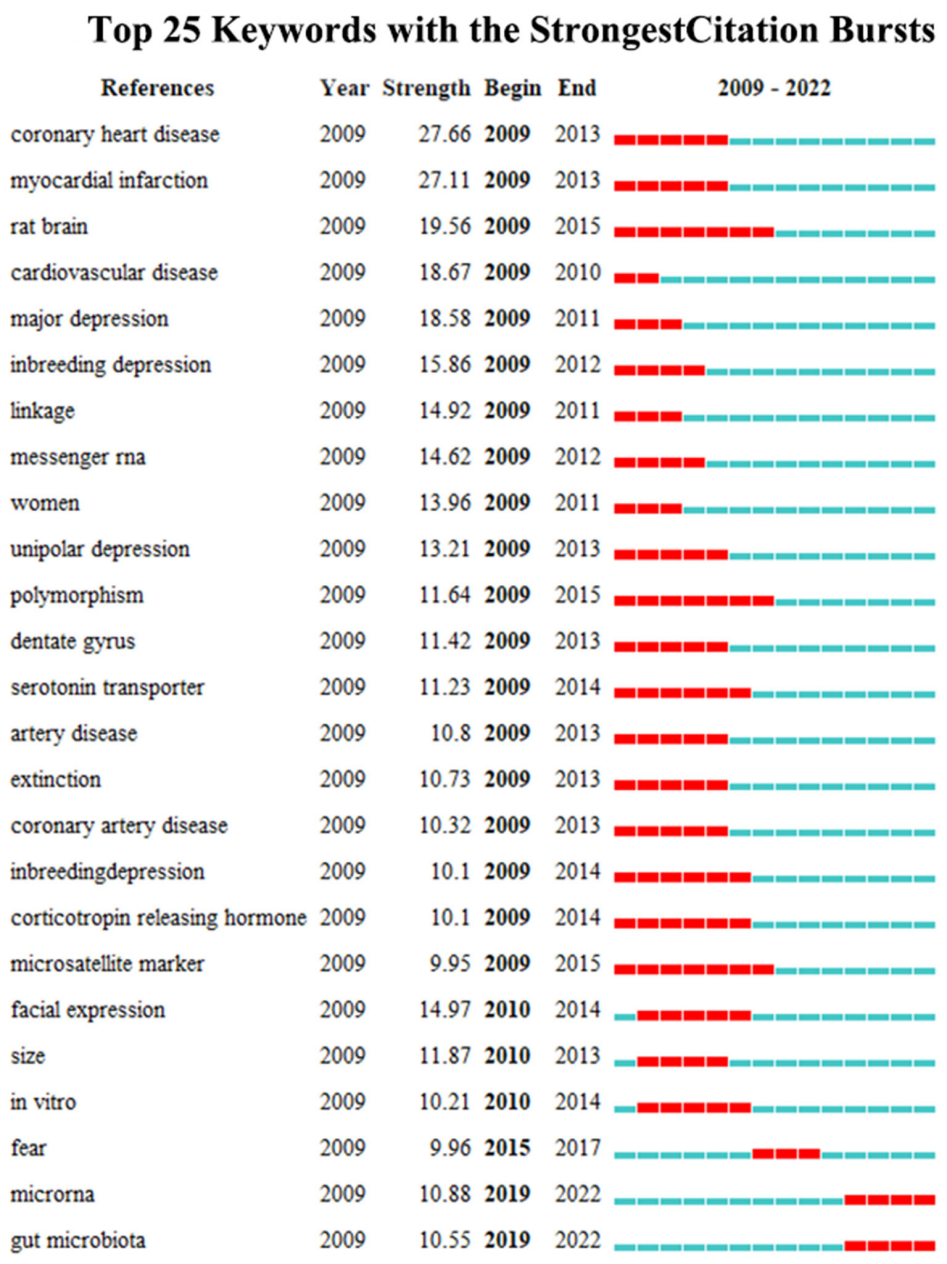
Top 25 keywords with the strongest citation bursts, 2009–2022.

Burst detection can reflect a sudden increase in citations for specific keywords and citations at a certain time, and accurately reveal hotspot evolution in the research field. The stronger the burst, the more attention the research issue has received and the more it reflects the research frontier of the period. In this study, we used CiteSpace to map the top 25 citations and keywords for biomarkers of depression from 2009 to 2022 based on the burst detection algorithm. Papers with high citation burst values can reflect emerging trends or themes within a field. CiteSpace's burst detection of citations indicated that Miller AH, Kohler CA, Drysdale AT, Goldsmith DR, Wray NR, Otte C, R Core Team, Malhi GS, and James SL have been especially active and maybe representative for cutting-edge research trends (Figure 7). “Coronary heart disease” had the largest keyword emergence value of 27.66, and “rat brain”, “polymorphism”, and “microsatellite markers” had the longest emergence times, all from 2009 to 2015. “MicroRNAs” and “gut microbiota” may be the frontiers of future research in this field (Figure 8).

## Discussion

With the development of modern social medicine, more attention has been paid to mental disorders. Depressive disorders, one of the most disabling mental disorders, continues to contribute to the global health-related burden today, especially in the post-COVID-19 pandemic. Due to the clinical and etiological heterogeneity, the pathogenesis of depression remains unclear. In the current study, we aimed to reflect research trends in the field of depression biomarkers by using a bibliometric approach in order to help researchers to quickly review and analyze research hotspots.

### Research hotspots

#### fMRI and machine learning

Functional magnetic resonance imaging (fMRI), which maps large-scale neural network function, particularly resting-state fMRI (rs-fMRI), has been suggested for classification of depression subtypes (22). Spontaneous signal fluctuations at rest measured by rs-fMRI are thought to represent functional connectivity (FC) between multiple brain regions, which can potentially be used as neuroimaging-based biomarkers (23). Functional connectivity is measured by correlating the activity time series of anatomically separate brain regions. Studies have shown that whole-brain rs-fMRI based on multivariate pattern analysis can distinguish patients with depression from healthy controls with 94.3% accuracy (24). Spontaneous fluctuations in resting-state FC in different brain regions may also be used to help clinicians optimize their treatment plans according to disease progression. The rostral anterior cingulate cortex (rACC) is located at the “hub” position in the default network, and studies have shown that pre-treatment rACC activity can be used as a marker of efficacy in patients with depression (25, 26). The volume of the subgenual anterior cingulate cortex (sgACC) has also been shown to be strongly associated with improvements in depressive symptoms after cognitive behavioral therapy (CBT) (27, 28). Monitoring the brain activity patterns of patients with depression by using fMRI can help clinicians predict treatment effects.

Machine learning is a method of learning from empirical data, building training models, and making accurate classifications using new data (29). The combination of machine learning and MRI data on depression can help us gain insights into the underlying neural circuitry of the brain. Support vector machines (SVM) is a common machine learning method currently used. An investigation of resting-state functional connectivity patterns in patients with depression showed that SVM can distinguish patients with major depression and healthy individuals accurately (94.3% accuracy and 100% sensitivity) (30). Machine learning also plays an important role in predicting the future progression of the disease and guiding individual-specific treatments efficiently. Redlich et al. explored whether neuroimaging techniques can predict the therapeutic effectiveness of electroconvulsive therapy (ECT). The results showed that the volume of the subgenual cingulate before treatment was positively correlated with the response to individual ECT and that binary pattern classification based on SVM could predict the response to ECT in depressed patients (78.3% accuracy, 100% sensitivity) (31). Machine learning is now widely used in the diagnosis of depression, but due to the relatively small sample size of studies and the heterogeneity of depression, accurate diagnosis of individuals based on neuroimaging remains difficult in clinical practice. The improvement of depression databases may strengthen the role of machine learning in depression diagnosis and prognosis in the future.

#### Inflammation, cytokines and oxidative stress

An increasing body of evidence indicates that aberrations in immune-inflammatory pathways and activation of cell-mediated immunity represent important pathophysiological pathways in the development of depression (32). Inflammatory cytokines have been shown to be associated with cognitive dysfunction in depression. It has been shown that peripheral cytokines can traverse the blood–brain barrier and act directly on neurons and supporting cells, such as astrocytes and microglia, or *via* signals mediated by afferent pathways, such as those in the vagus nerve, to activate neuroinflammation (33). Moreover, activation of inflammatory signaling pathways could lead to disturbances in the regulatory networks of the neuroendocrine, monoaminergic, neurotropic, and hypothalamic-pituitary-adrenal (HPA) axes, which in turn are involved in the development of depression (34). It has been shown that the inflammatory factors IL-1β, IL-6, TNF, and C-reactive protein (CRP) in peripheral blood are reliable biomarkers for patients with depression (35). In addition, genetic polymorphisms in inflammatory factors (e.g. IL-1β, TNF, and CRP) are strongly associated with depression and treatment outcomes (36). With an increased understanding of how inflammatory processes can lead to depression, various classes of anti-inflammatory medications have come under consideration. For example, the inhibition of neuroinflammatory cytokines (such as TNF or cyclooxygenase 2) has been shown to have significant antidepressant effects in depressive patients with rheumatoid arthritis, psoriasis, or cancer (37). Inflammation is associated with depression, and the degree of inflammation can affect depressive symptoms. Utilizing the degree of inflammation to achieve patient-specific treatment is a challenge for future research.

Previous studies have reported increased levels of inflammatory cytokines in the peripheral blood of patients with depression. These inflammatory cytokines can access the brain and interact with pathophysiological mechanisms in depression (e.g., neurotransmitter metabolism, neuroendocrine function, and neural plasticity) (38). The efficacy of antidepressant therapy is also affected by increased neuroinflammatory factors; Therefore, targeting inflammatory factors and signaling pathways in the nervous system has become a new strategy for the treatment of depression. A study by Abbasi et al. showed that celecoxib, a drug that selectively targets COX-2, inhibits inflammatory factors in tissues, particularly interleukin (IL)-6 and IL-1, thereby promoting recovery and reducing the risk of relapse in patients with depression (39). Similarly, other cytokine inhibitors have shown good therapeutic efficacies in clinical trials. In patients with baseline hs-CRP > 5 mg/L, a monoclonal antibody drug directed at TNF-α (infliximab) can significantly reduce depressive symptoms, including depressed mood, anxiety, and suicidal ideation (40). Unfortunately, among anti-inflammatory strategies, only patients with depression and high initial inflammation or high baseline CRP levels showed significant antidepressant effects (37). Targeting TNF-α inhibition in patients with normal cytokine expression levels may be counterproductive and exacerbate depressive symptoms. Therefore, it is important for clinicians to pay attention to inflammatory factor levels during personalized antidepressant treatment. In addition, since the expression levels of inflammatory cytokines vary greatly, clarifying the pattern of inflammatory expression in patients with depression is the next direction of work. The combination of multiple biomarkers should also be considered to explore the best therapeutic targets for anti-inflammatory interventions.

Oxidative stress is an imbalance between oxidants and antioxidants, resulting in the disruption of redox signaling and molecular damage (41). Studies have confirmed increased oxidative stress and reduced antioxidant capacity in patients with depression (42). Under pathological conditions, oxidative stress may induce neurodegeneration *via* various pathways such as the induction of apoptosis, excitotoxicity, and axonal damage (43). The levels of reactive oxygen species (ROS), antioxidants, and antioxidant enzymes can reflect the body's redox homeostasis, and these markers have been extensively studied in psychiatric disorders. A recent meta-analysis showed that F2-isoprostane and 8-OHdG (which mediate lipid oxidation and oxidative DNA damage, respectively) are the two most consistently elevated oxidative stress markers in patients with depression. Moreover, Abdel-Wahab and Salama showed that long-term administration of antidepressant doses of venlafaxine significantly reduced serum and hippocampal levels of 8-OHdG in stressed animals, suggesting potential antioxidant effects associated with these antidepressants (44). Cumurcu et al. found significantly higher levels of total oxidant status (TOS) and oxidative stress index (OSI) and lower levels of total antioxidant capacity (TAC) in patients with major depressive disorder (MDD) compared with controls, and the size of the deviations was correlated with disease severity. After 3 months of treatment with selective serotonin reuptake inhibitors, TOS and OSI expression was significantly reduced and TAC expression was increased (45). These studies suggest that antidepressants may exert neuroprotective effects *via* antioxidant defense mechanisms in patients with depression. Current methods for detecting levels of oxidative stress are mainly based on assays of circulating body fluids (e.g., blood and urine); however, biomarkers of peripheral circulation may not reflect changes in the central nervous system. Subsequent studies should therefore focus on the specific role of oxidative stress in the central nervous system.

#### Brain-derived neurotropic factor

Brain-derived neurotropic factor (BDNF) is a neurotrophic peptide essential for axonal growth, regulation of neuronal survival, and synaptic remodeling (46). Karege et al. first demonstrated that serum BDNF levels were lower in patients with depression than in healthy individuals (47). Subsequent clinical studies assessing BDNF levels in patients with depression have identified an important correlation between depression and BDNF levels, finding that increased BDNF levels are a key indicator of antidepressant effectiveness (48). The neurotrophic factor hypothesis suggests that stress-induced reductions in BDNF expression may lead to atrophy of the hippocampus and prefrontal cortex, which in turn may lead to depression. Conversely, antidepressants may exert a therapeutic effect by restoring central BDNF activity. Therefore, BDNF is considered to be a diagnostic biomarker that can be used to predict the efficacy of antidepressants. Karlovic et al. found that serum BDNF could also be used as a candidate marker to distinguish healthy individuals from those with depression, and the receiver operating characteristic (ROC) curve for BDNF that they constructed had a diagnostic sensitivity of 83.9% and specificity of 93% at a threshold concentration of 48.1 ng/ml (49). These results suggest that BDNF levels in the peripheral blood may be a valuable diagnostic biomarker for clinical assessment. However, reduced plasma or serum levels of BDNF are not specific to depression, and the same results have been observed in other mental disorders, such as schizophrenia, bipolar disorder, and Alzheimer's disease. This lack of specificity limits the use of BDNF for clinical tests. Therefore, studies combining BDNF with other diagnostic markers should be conducted.

#### Metabolomics

Metabolomics, as a novel “omics” approach, helps identify metabolic features or biomarkers of specific diseases by qualitatively and quantitatively analyzing small-molecule metabolites to reflect the downstream effects of individual environmental, genomic, and proteomic variations (50). This technique has been widely used in mechanistic studies of depression, and the key metabolites measured in related studies may be potential diagnostic and prognostic biomarkers of depression. Most key metabolites and their associated pathways in the pathology of depression focus on three main topics: lipid and fatty acid metabolism, amino acid metabolism, and glucose and energy metabolism. Analysis of several molecular indicators of lipids confirmed that increased levels of total cholesterol and triglycerides (TG) and decreased levels of low-density lipoprotein cholesterol (LDL), high-density lipoprotein cholesterol (HDL), and u-3 polyunsaturated fatty acids in the peripheral blood of patients were associated with depression (51). Amino acid metabolism is involved in the pathogenesis of depression by affecting neurotransmission in the brain, particularly the persistent downregulation of GABA due to glutamate imbalance and disruption of the 5-hydroxytryptaminergic system due to reduced tryptophan levels (52). Metabolites involved in carbohydrate and energy metabolism, such as glucose, lactate, pyruvate, malonate, methylmalonate, and succinyl-CoA, are expressed at altered levels in patients with depression (53). Although metabolomic studies have identified multiple candidate metabolites relevant to depression and therapeutic responses, only a few metabolites (including u-3 polyunsaturated fatty acids) have been validated in patient cohort studies. Therefore, the translation of metabolic biomarkers into clinical indicators for the diagnosis or treatment of depression remains a major challenge.

#### MicroRNAs

In recent years, a growing number of studies have confirmed the role of non-coding RNAs in depression. MicroRNAs (miRNAs) are among the most researched and well-characterized noncoding RNAs related to depression. miRNAs are single-stranded RNA molecules (17–22 nucleotides) that regulate mRNA expression by degrading RNA or inhibiting protein translation (54). Each miRNA can modulate the expression of multiple genes, enabling them to regulate multiple cellular signaling pathways. In 2012, Belzeaux et al. first explored miRNA expression profiles in patients with depression and showed that 14 miRNAs differed in peripheral blood samples from patients with depression compared with those from healthy controls (including miR-941, miR-376a-5p, miR-589, miR-1267, miR-331-5p, miR-100-3p, miR-342-5p, miR-571, let-7b, miR-454, hsa-miR-345, miR-33a-3p, miR-363, and miR-331-3p) (55). In addition, studies of RNA-Seq of blood samples from patients with MDD has also revealed that the miR-let-7 and miR-34 families show significantly changed expression in depressive patients and have the potential to be diagnostic biomarkers for depression (56). miRNAs also play an important role in antidepressant treatment. The results of a large randomized placebo-controlled trial suggest that miR-146a-5p, miR-146b-5p, miR-24-3p, and miR-425-3p are potential prognostic markers for depression treatment. Moreover, these findings were replicated in animal models of depression and in post-mortem human brains (57). In summary, owing to their non-invasive nature and stability, miRNAs have been considered ideal diagnostic biomarkers for depression, as well as potential therapeutic targets and promising prognostic biomarkers. However, for clinical applications, the identification of several miRNAs as unique and reliable biomarkers for depression is unrealistic. A more effective clinical strategy may be the development of a portfolio of biomarkers based on molecular, imaging, and clinical data.

### Frontiers of research

The citation burst value of an article represents citation frequency and may indicate the level of innovation a study provides and marks the cutting edge of research in this field during that period. The top 10 citations with high burstiness reflect both the hot research issues of this discipline in the corresponding time interval and reveal future research trends in this discipline. Therefore, we will discuss the bursting of papers in this field up to 2022. Otte, Malhi, and James provide detailed reviews of the current state of research on depression, summarizing current epidemiology, pathophysiological mechanisms of depression, and its diagnosis and treatment. Miller's study argues for a pathophysiological mechanism of neuroinflammation in the development of depression and suggests that anti-inflammatory therapy may be a potential strategy for future antidepressant treatment (35). Goldsmith and Kohler, on the other hand, have studied cytokines and chemokines in depressed patients and speculated about possible causes of depression heterogeneity, suggesting that cytokines and chemokines in peripheral blood can be used as diagnostic biomarkers for major depressive disorder (32, 58). Drysdale et al. noted that functional connectivity studies based on functional magnetic resonance imaging (fMRI) can be used to classify subtypes of depression with extremely high sensitivity (82–93%) and specificity. fMRI is a good diagnostic classifier and holds promise for addressing the difficult issue of heterogeneity in depression (6). Wray used a large-scale genome-wide association study to identify genetic risk factors for depression, and identified 44 separate loci that were strongly associated with depression risk (59). In summary, neuroinflammation, cytokines, functional magnetic resonance imaging, and genetic variants may be at the forefront of future research on depression biomarkers.

Today, we are moving into a new phase of the pandemic, COVID-19 has impacted research and scientific publications in almost all fields, including the field of psychiatry and mental health. Santomauro D F et al. suggested a substantial increase in the prevalence and burden of major depressive disorder and anxiety disorders as a result of the COVID-19 pandemic (60). In such a context, schizophrenia, mental health and depression are gradually becoming popular publication topics. Unfortunately, although this bibliographic analysis study included post-pandemic literature, no association between depression biomarkers and COVID-19 was found in the results of the co-word and co-citation analyses. This observation may be related to our search strategy, which focused on biomarkers of depression and aimed to explore indicators of depression etiology and clinical relevance. Previous studies have suggested that the contribution of COVID-19 to the increased prevalence of depression has been attributed to population shocks, social isolation, quarantines and uncertainty about the future (60). It has also been suggested that the association of COVID-19 with depression is inextricably linked to neuroinflammation (61, 62). da Silva Lopes L et al. noted that due to the action of inflammatory cytokines and the presence of cell surface ACE-2 receptors, COVID-19 survivors may be more susceptible to depression (63). Therefore, further studies are needed to elucidate the association between SARS-CoV-2 infection and depressive disorders.

This study has some limitations that may be overcome in future bibliometric studies. First, the data were obtained from the WoSCC database. However, other databases (e.g., PubMed, Google Scholar, Baidu Scholar, Scopus, and EMBASE) may contain wider and more in-depth coverage of the literature. In this study, secondary information literature such as reviews and editorials were omitted. Second, the multi-author, multi-institutional situation is not well managed and also neglects the influence of economic and demographic circumstances between different countries/regions on relevant research inputs, which may be potentially biased. Third, due to 2022 is still ongoing, the overall data of 2022 cannot be included. These omissions may produce research bias. Nevertheless, this study adds to our knowledge of hotspots and cutting-edge trends in depression biomarker research, and will benefit future antidepressant treatment research.

## Conclusion

In this article, we present a comprehensive overview of the knowledge structure of depression biomarkers through co-word and co-citation analyses. Eight clusters of depression biomarker research were obtained using bibliometric analysis, and a review of their research progress was presented. Among these research hotspots, functional magnetic resonance imaging (fMRI), cytokines, and oxidative stress are relatively well established, whereas research on machine learning, metabolomics, and microRNAs is still immature and should be considered future trends in this area. Further, miRNAs and gut microbiota are still hot research topics and are likely to remain a focus of future research. Further analysis of these research topics may help improve our knowledge of the biomarkers of depression and provide guidance for future treatment.

## Data availability statement

The original contributions presented in the study are included in the article/Supplementary materials, further inquiries can be directed to the corresponding author.

## Author contributions

X-jG and YR conceived of the study and designed the study. PW and XJ analyzed the data and wrote the initial draft of the manuscript. Y-mD, C-mZ, N-nC, and C-rG processed the figures and tables. All authors listed have made a substantial, direct, and intellectual contribution to the work and approved it for publication.

## Funding

This work was supported by the National Natural Science Foundation of China (8210053813), Applied Basic Research Projects of Shanxi Province (201901D111418), Research Project Supported by Shanxi Scholarship Council of China (2021-167), and Basic Research Priorities Program of Shanxi Province (20210302123314).

## Conflict of interest

The authors declare that the research was conducted in the absence of any commercial or financial relationships that could be construed as a potential conflict of interest.

## Publisher's note

All claims expressed in this article are solely those of the authors and do not necessarily represent those of their affiliated organizations, or those of the publisher, the editors and the reviewers. Any product that may be evaluated in this article, or claim that may be made by its manufacturer, is not guaranteed or endorsed by the publisher.
