# Supplementary material for: Mapping the structure of depression biomarker research: A bibliometric analysis
Source: Front Psychiatry. 2022 Sep 16;13:943996. doi: 10.3389/fpsyt.2022.943996 (PMC9523516; doi:10.3389/fpsyt.2022.943996)
Supplement: Supplementary file 1 [file Data_Sheet_1.pdf]

Mapping the Structure of Depression Biomarker Research: A Bibliometric Analysis

1 The Core Authors and Intellectual Basis of the Research Field

To identify scholars with a large influence in the field of depression biomarkers and to gain insight into the most influential or foundational knowledge in the field, we constructed an author co-citation collaboration network. In the co-citation network, the size of the nodes represents the frequency with which the author's work has been cited, and the thickness of the lines between nodes represents the number of co-citations of connected authors.

Since 2009, 72,374 authors have contributed to the research on biomarkers of depression. Of these authors, the top 10 have been involved in the publishing of a total of 1270 articles (8.82%). Maes M had the highest number of articles, with 89 (0.62%), and Penninx B.W.J.H. had the second-highest number of articles, with 88 (0.61%). Figure 4 shows the co-citation network, and Table 3 lists the top 10 cited authors. The results showed that Maes M ranked first with 2673 citations, Berk, M second with 2607, and Penninx ranked third with 2379 citations.

Supplementary Table 1 The top 10 most procreative authors who contributed to publications and the top 10 co-cited authors and references in depression biomarker research

Rank	Author	Frequency	Co-cited Authors	Frequency	Top 10 References	Frequency
1	Maes M	89	Maes M	2673	10.1016/S0140-6736(12)62129-1	1840
2	Penninx BWJH	88	Berk M	2607	10.1001/2013.jamapsychiatry.4	927
3	Wang Y	80	Penninx BWJH	2379	10.1016/j.bbi.2015.03.016	861
4	Berk M	70	Zarate C	2274	10.1038/nm.4246	816
5	Zhang Y	70	Mayberg H	2250	10.1001/archpediatrics.2009.214	702
6	Teixeira AL	67	Miller A	2102	10.1056/NEJMoa1001593	698

7	Li Y	60	Kapczinski F	1919	10.1016/j.marenvres.2012.04.003	623
8	Kennedy SH	49	Irvin M	1884	10.1093/bioinformatics/btu848	589
9	Liu Y	48	Xie P	1821	10.1038/mp.2012.105	572
10	Hickie IB	45	Phillips M	1756	10.1016/j.landurbplan.2011.12.015	533

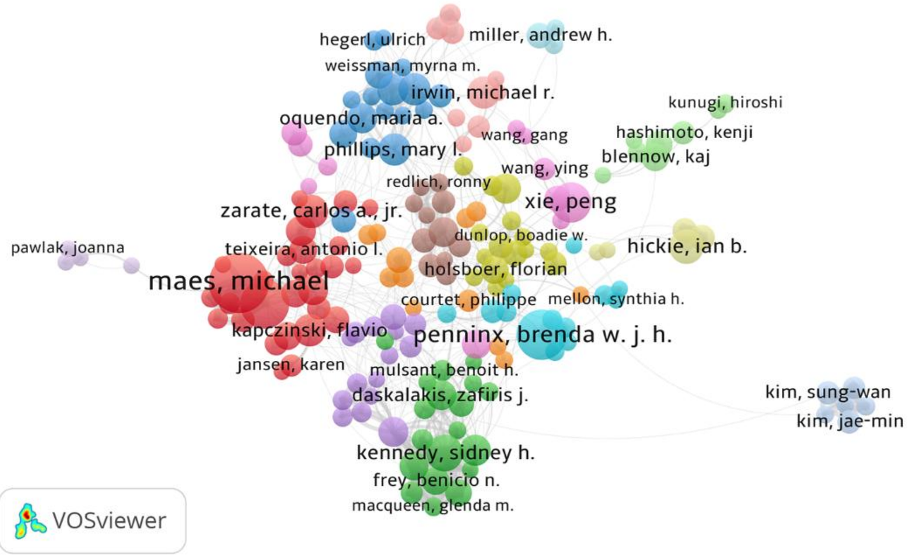

Supplementary Figure 1 The visualization of co-citation network mapping of authors in depression biomarker research. The thickness of the lines indicates the strength of the relationship.

2 Clustering Analysis of Keywords and Co-word Analysis

Keywords serve as a comprehensive overview and distillation of a paper's content and subject matter. Keyword co-occurrence is commonly used to reveal research hotspots. Cluster and hotspot analyses of keywords based on VOSviewer were conducted to identify the main research directions and knowledge hotspots for depression biomarkers. The results are shown in Figure 5. The node size and colour indicate the number of keywords and clusters, respectively. The co-occurrence frequency between keywords is indicated by the thickness of the line between the nodes, and different clusters represent different research directions and hotspot areas. The closer the colour is to yellow in the hotspot density plot, the higher the frequency of keywords in that area, which reveals the research hotspots in this field.

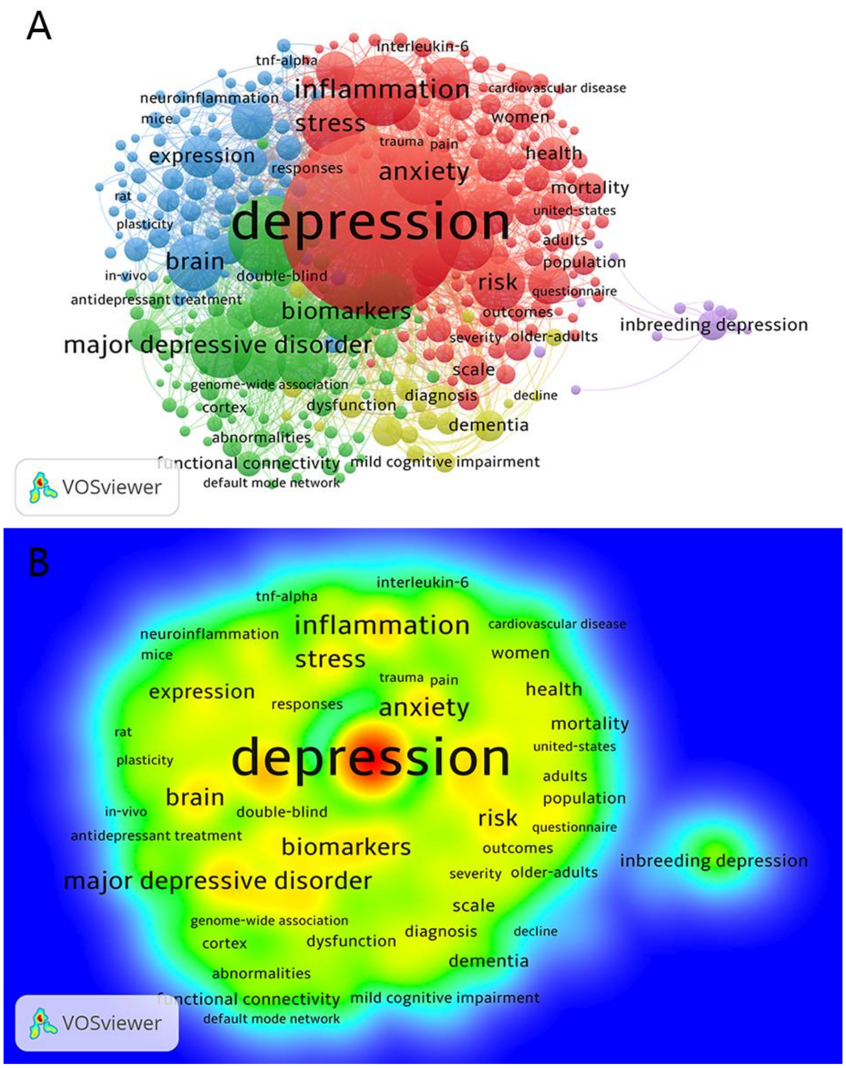

Supplementary Figure 2 The visualization of keywords co-occurrence analysis in the depression biomarker research. (A) Keyword Co-occurrence Map Based on VOSviewer; (B) Keyword Density Map based on VOSviewer.

3 Similarity between depression and psychosis

In addition, we conducted a literature search in WOSCC using the strategy "(Psychosis *) AND "(biomarker *) or (biomarkers *) or (marker *) or (markers *)" and restricted the type of literature to 'article' and the language type to 'English', which resulted in 2406 articles related to psychiatric biomarkers. These documents were then downloaded in txt format and imported into Vosviewer and Citespace for keyword co-occurrence network and reference co-citation analysis (the same methods we used in the main text).

The keyword clustering results show that the research themes of biomarkers of psychosis are divided into five clusters. In the first cluster (red), 'schizophrenia' appears most frequently as the primary keyword, followed by 'psychosis', 'affective disorders', 'biomarkers', 'depression', 'inflammation' and 'cytokines'. In the second cluster (green), the most frequently used keyword in this cluster is 'stage 1 schizophrenia', with other high-frequency keywords including 'abnormal conditions', 'brain', 'prefrontal' and 'functional connectivity'. In the third cluster (blue), the keyword 'cognition' appears most frequently, followed by 'deficit' and 'dysfunction'. The fourth cluster (yellow) includes 'stage 1 psychosis', 'clinical high risk' and 'predicted'. The fifth group (purple) contains only three keywords, including 'adolescent', 'child' and 'mental disorder'. The reference co-citation results show that articles published between 2001 and 2021 related to psychiatric biomarkers were grouped into seven clusters, including inflammation, vbm, transdiagnostic, high risk, hippocampus, depression and mismatch negativity.

In this preliminary comparative analysis, multiple high-frequency keywords were present with both studies, demonstrating some association between the two brain disorders. Similarly, the RCA analysis showed similar findings, with inflammation and imaging-related labels found in the psychosis study hotspot clusters. As the subject of this study is biomarkers of depression, it is hoped that other brain disorders can be explored further in future follow-up studies.

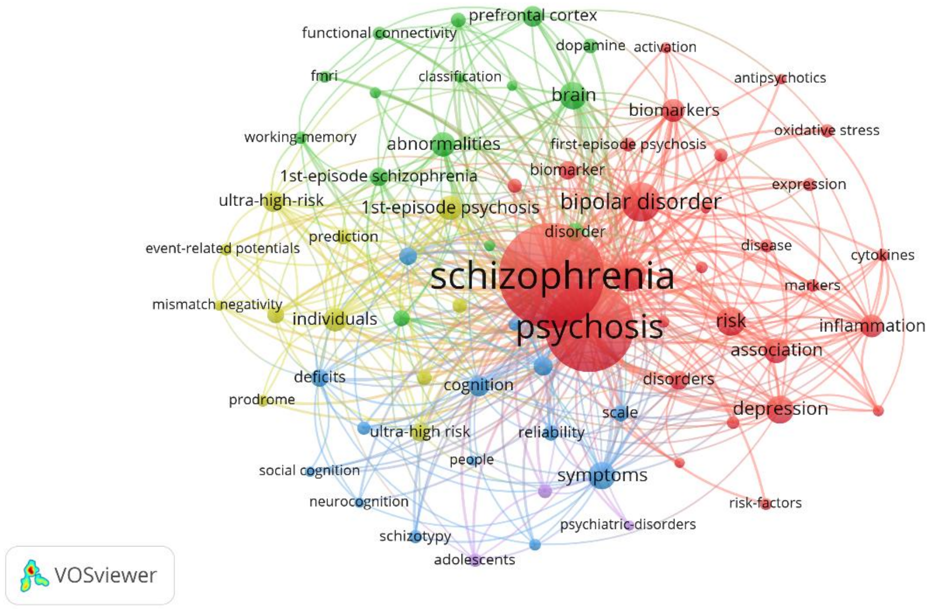

Supplementary Figure 3 The visualization of keywords co-occurrence analysis in the psychosis biomarker research.

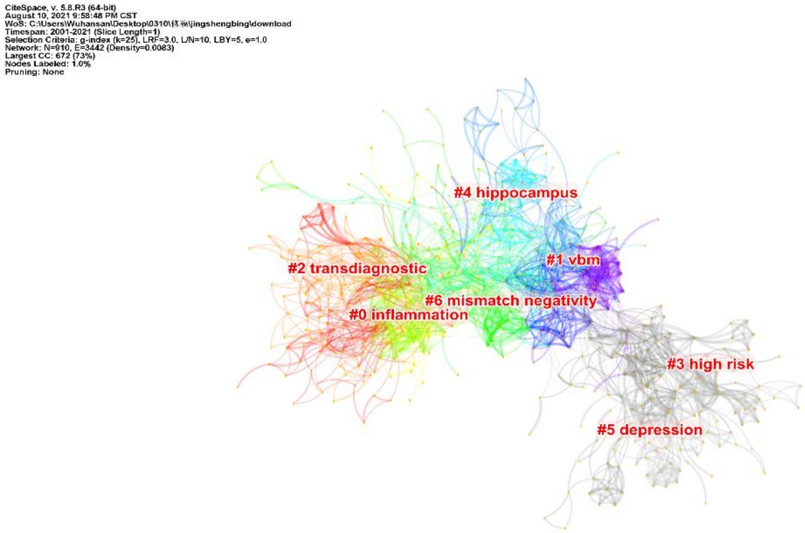

Supplementary Figure 4 The visualization of reference co-citation analysis of psychosis biomarker research.
